# Utilization of Industrial Hemp Biomass Waste (I): Stability of Cannabidiol in Pre and Post- Encapsulation States

**DOI:** 10.3390/molecules30102116

**Published:** 2025-05-10

**Authors:** Jerel Crew, Ying Wu, Richard Mu, Ankit Patras

**Affiliations:** 1 Department of Food and Animal Sciences, Tennessee State University, Nashville, TN 37209, USA; jcrew@tnstate.edu (J.C.); apatras@tnstate.edu (A.P.); 2 Department of Biological Sciences, Tennessee State University, Nashville, TN 37209, USA; 3 TSU Interdisciplinary Graduate Engineering Research (TIGER) Institute, Tennessee State University, Nashville, TN 37209, USA; rmu@tnstate.edu

**Keywords:** CBD, hemp biomass, thermal stability, photostability, encapsulation

## Abstract

After cannabidiol was extracted from the hemp biomass using supercritical CO_2_ extraction, the residual could be utilized as a source of other valuable ingredients. The stability of the extracted CBD in pre- and post- encapsulation states were evaluated. Dynamic macerations with ethanol and hexane were compared for CBD extraction. The ethanol extract yielded 0.11% ± 0.10 CBD and 1.83% ± 0.00 cannabidiolic acid (CBDA), while the hexane extraction yielded 0.08% ± 0.04 CBD, 1.06% ± 0.04 CBDA, and 0.30% ± 0.04 delta-9-tetrahydrocannabinol (Δ9-THC). Ethanol extraction was selected due to the low THC detection in the extract. The CBD extract was encapsulated using water soluble yellow mustard mucilage (WSM), maltodextrin (MD), gum Arabic (GA), and protein extracted from the hemp biomass waste (HBP) via freeze drying. The WSM-MD-GA 1:5 particle formulation exhibited superior thermal stability over 72 h, whereas the WSM-HBP-GA 1:5 formulation offered the most protection against UVa-induced degradation within the same duration. Incorporating hemp biomass protein as an encapsulation material enhanced protection against light exposure through UV absorption, although it did not grant thermal protection. These findings indicated that encapsulation significantly protects against CBD degradation when subjected to thermal and light conditions compared to non-encapsulated CBD.

## 1. Introduction

Industrial hemp, a variety of the *Cannabis sativa* plant species, has been cultivated for thousands of years for its fiber, oil, and seeds. It is used in various products ranging from textiles and biofuels to food and dietary supplements. In recent years, interest in industrial hemp has expanded beyond its traditional uses to the exploration of its cannabinoids and bioactive compounds with significant therapeutic potential. Among these, cannabidiol (CBD) has garnished a lot of interest for its non-psychoactive properties and potential health benefits, including anxiolytic, analgesic, and anti-inflammatory effects [1].

The increasing demand for sustainable and biodegradable materials leads to the exploration of natural polymers for various applications, including food and drug delivery systems. Natural polymers, such as proteins, polysaccharides, and lipids, offer advantages over synthetic polymers regarding lower toxicity, biodegradability, and biocompatibility [2,3]. Among natural polymers, hemp protein, derived from industrial hemp biomass, including leaves, flowers, and stems, presents an intriguing option due to its favorable amino acid profile, environmental sustainability, and comparable physicochemical properties with other commercial products.

The encapsulation of industrial hemp-derived cannabinoids using natural polymers, including hemp protein, represents a novel approach that combines the therapeutic potential of cannabinoids with the health benefits of natural polymers. Encapsulation can enhance the stability of cannabinoids, addressing some of the major challenges in their delivery and effectiveness. Furthermore, the use of hemp protein as an encapsulating material not only aligns with the principles of sustainability and waste minimization but also adds value to hemp biomass waste by utilizing its by-products.

The extraction and isolation of cannabinoids span from traditional techniques such as dynamic maceration (DM) [4], Soxhlet extraction, and percolation to more non-traditional approaches like microwave-assisted extraction (MAE), ultrasound-assisted extraction (UAE), and supercritical fluid extraction (SFE). These methods are differentiated by their mechanisms and efficiency in yielding cannabinoids [5]. In the current study, the hemp biomass waste was obtained from the residue after supercritical CO_2_ extraction. Due to the large-scale processing and coarse particle size, there is always a certain amount of CBD remaining in the residue. To further extract the remaining CBD, the dynamic maceration [4] was selected for its cost-effectiveness and readily accessibility. This method involves further extracting the remaining compounds from finely grinded biomass using a solvent, coupled with constant stirring at ambient temperature. DM has been identified as particularly effective in extracting cannabidiolic acid (CBDA) compared to other techniques [6]. The efficacy of CBD extraction can be significantly influenced by the solvent utilized. Cannabinoids exhibit solubility in non-polar and polar solvents [7]. The solvent used for the extraction determines not only the type but also the yield of cannabinoids extracted [8].

This study aims to further utilize hemp biomass waste through the extraction of the remaining CBD, and to examine its stability in both pre- and post-encapsulation states when encapsulated with natural polymers.

## 2. Results

A total of 11 compounds, cannabidivarin (CBDV), cannabigerol (CBG), cannabigerolic acid (CBGA), cannabidiolic acid (CBDA), cannabidiol (CBD), tetrahydrocannabivarin (THCV), cannabinol (CBN), delta-9-tetrahydrocannabinol (Δ9-THC), delta-8-tetrahydrocannabinol (Δ8-THC), cannabichromene (CBC), and tetrahydrocannabinolic acid (THCA), were employed as reference standards for the CBD profiling. Three major peaks were depicted from the HPLC chromatography, namely CBDA, CBD, and Δ9-THC.

### 2.1. Comparison of Solvents for Cannabinoid Extraction

**Figure 1:**
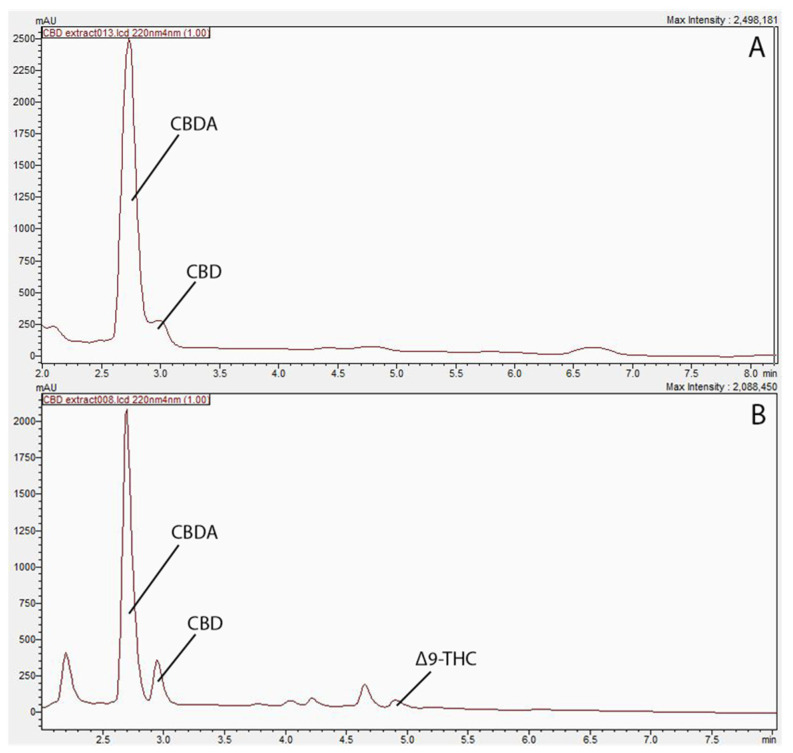
Profiling of cannabinoids in biomass residue post supercritical carbon dioxide extraction. (**A**) Chromatogram of cannabinoid profile in ethanol extract at 220 nm. (**B**) Chromatogram of cannabinoid profile in hexane extract 220 nm.

The concentrations of extracted compounds using hexane and ethanol are presented in Table 1, and their corresponding HPLC chromatographs were presented in Figure 1 as well. As shown Figure 1, the ethanol extract exhibited two major peaks, identified as CBD and CBDA, while the hexane extract has displayed extra peaks in the 4–5 min retention time range, and were identified as Δ9-THC. Similar results were obtained by Moreno-Sanz et al. [7], who have also extracted hemp biomass and found more compounds emerged using hexane. The results revealed the presence of CBD, CBDA, and Δ9-THC within the hexane-extracted samples, while the ethanol-extract showed peaks of CBD and CBDA only. The difference in polarity between the two solvents supports this inconsistency in cannabinoid yield. Specifically, the non-polar nature of hexane is speculated to extract non-polar cannabinoids, such as THC, more efficiently in contrast to the polar solvent ethanol [9]. Since CBD was the compound of interest in the current study, ethanol extraction was adopted for further study to obtain adequate CBD oil for this research.

### 2.2. Stability Study of CBD Oil in Pre- and Post-Encapsulation Status

Stability of CBD was measured using free and encapsulated CBD. For encapsulated CBD, core-wall ratios of 1:1, 1:2, and 1:5 (CBD extract to wall ingredients) were compared. Water soluble yellow mustard mucilage (WSM), maltodextrin (MD), and Gum arabic (GA) were used at the ratio of 1:2:5, respectively. The formula was proven with superior controlled release properties due to the core ingredients being protected by the wall matrix [10]. Protein extracted from the biomass waste used in the current study was also tested as an encapsulation ingredient for the core–wall ratio of 1:5 formula, where dextrin was replaced by the biomass protein (HBP). The initial CBD content in free and encapsulated samples was expressed as 100%. The CBD content after thermal and photo treatments were compared with the initial CBD content and expressed as a percentage (%) of the initial CBD content.

#### 2.2.1. Thermal Stability Study

**Figure 2:**
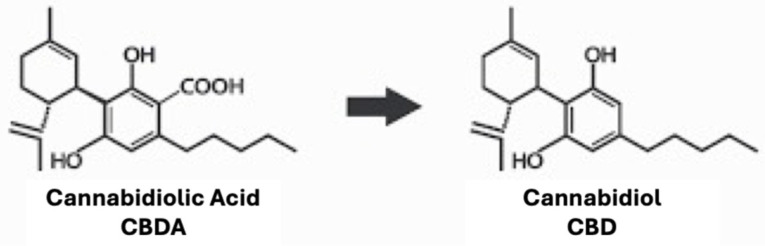
Decarboxylation process from cannabidiolic acid (CBDA; **left**) to cannabidiol (CBD; **right**).

In the encapsulated and nonencapsulated samples, CBDA was the most prevalent cannabinoid detected. Exposure to elevated oven temperatures for 72 h caused the cleavage of carboxyl groups in CBDA, leading to an increase in CBD content, as shown in Figure 2. CBD degradation related to elevated temperatures was also observed.

**Figure 3:**
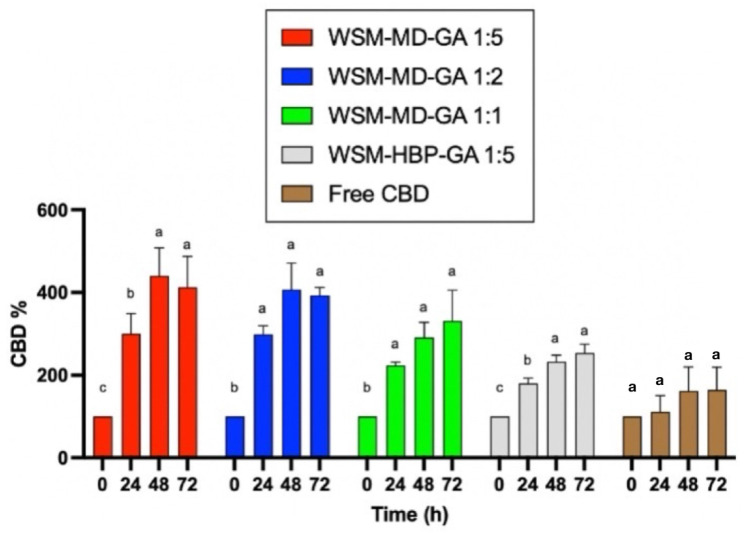
Comparative analysis of the thermal degradation of encapsulated versus non-encapsulated CBD over time intervals of 0, 24, 48, and 72 h under elevated temperature conditions (70 °C). Data are expressed as a mean ± STDEV. Significant differences within the encapsulation groups were determined using Tukey’s test. ^a,b,c^ Data indicated with different letters differ significantly (*p* < 0.05).

Figure 3 displays the change in CBD concentration during thermal treatments among encapsulated versus free CBD over 72 h at the elevated temperature condition (70 °C). Notably, the formulation WSM-MD-GA 1:5 exhibited the highest resistance of CBD to change. This formulation exhibited the maximal retention of CBD across all examined time intervals, with an observed increase in concentration at the 48 h benchmark. Such efficacy is attributed to the optimal 1:5 core-to-wall ratio, which establishes a durable protective shell around the CBD. The large increase in CBD was attributed to the conversion from CBDA to CBD. As shown in Table 1, the amount of CBDA is more than 10 times that of CBD in the extract.

Formulation WSM-HBP-GA 1:5 was susceptible to thermal degradation compared to the other encapsulated CBD groups. Within this composition, the retention of CBD was minimally observed across various time intervals, resulting in a peak increase in CBD concentration after 72 h.

All encapsulated CBD groups showed better retention of CBD compared to the free CBD groups. This phenomenon can be attributed to the interactions among hemp biomass protein, WSM, and gum Arabic. The replacement of maltodextrin (MD) with hemp protein (HBP) in this formulation resulted in an insufficient protective outer layer, thereby making the core compound more vulnerable to thermal exposure.

#### 2.2.2. Photo Stability Study

**Figure 4:**
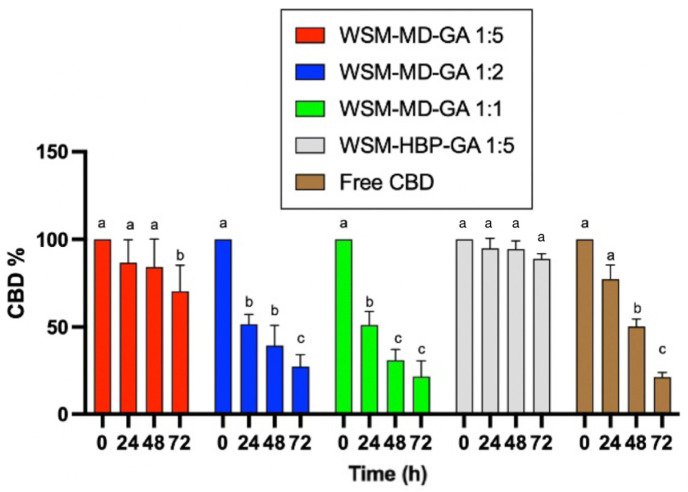
Comparative analysis of photodegradation in encapsulated versus non-encapsulated CBD across time intervals of 0, 24, 48, and 72 h under UVa light exposure. Data are expressed as a mean ± STDEV. Significant differences among the groups were determined using Tukey’s test. ^a,b,c^ Data indicated with different letters differ significantly (*p* < 0.05).

This experiment assessed the photostability of both encapsulated and non-encapsulated CBD under the influence of ultraviolet A (UVa) light (365 nm) at ambient temperature (25 °C). Figure 4 compares encapsulated versus non-encapsulated CBD degradation due to UVa exposure over 72 h. Consistent with expectations, the encapsulated CBD exhibited the least degradation when exposed to UV light compared to its non-encapsulated counterpart.

The degradation of free CBD was significant after 48 h. Only around 25% of CBD remained after 72 h of treatment. A similar trend was observed for the encapsulated CBD for the core–wall ratios of 1:1 and 1:2, indicating that the amount of wall materials did not provide protection over UVa degradation. The core–wall ratio of 1:5 provided better protection over CBD degradation against UVa treatment. The formulation WSM-HBP-GA 1:5 showed superior protection effect over CBD degradation, with no significant change in CBD observed after 72 h exposure to UVa light.

## 3. Discussion

### 3.1. Comparison of Solvents for Cannabinoid Extraction

This experiment performs a comparative analysis of the cannabinoids extracted from hemp biomass waste utilizing polar and non-polar solvents, specifically ethanol and hexane. Table 1 displays the cannabinoid yields obtained through dynamic maceration [4] using ethanol compared to hexane. As outlined in Table 1, the application of DM with hexane and ethanol resulted in distinct yields of cannabinoids per gram of hemp biomass. The most abundant cannabinoid isolated, irrespective of the solvent used, was CBDA, extracted at 10.65 and 18.89 mg/g for hexane and ethanol, respectively. Conversely, CBD was identified as the least abundant cannabinoid, extracted at 0.87 and 1.18 mg/g for hexane and ethanol, respectively. Among the profile of cannabinoids extracted, ethanol facilitated a higher yield of desired cannabinoids (CBDA, CBD); in contrast, hexane yielded a lower yield. A prior study investigating cannabinoid yields employing ethanol and hexane through DM validated these findings [6]. Furthermore, when evaluating different extraction methodologies, ethanol used in conjunction with DM was observed to extract a superior yield of cannabinoids relative to supercritical fluid extraction (SFE) or ultrasonic-assisted extraction (UAE) [11].

### 3.2. Thermal Stability

Conversely, research conducted by Wang et al. (2022) [12] highlighted that whey protein could significantly enhance the thermal protection of core compounds encapsulated within zein particles. This enhanced protection was attributed to the formation of a denser shell enabled by the interactions between whey protein and zein. Such findings suggest that the inverse effect may be observed in the WSM-HBP-GA particles, where the specific interaction between hemp protein, WSM, and gum Arabic does not grant the same level of thermal protection.

**Figure 5:**
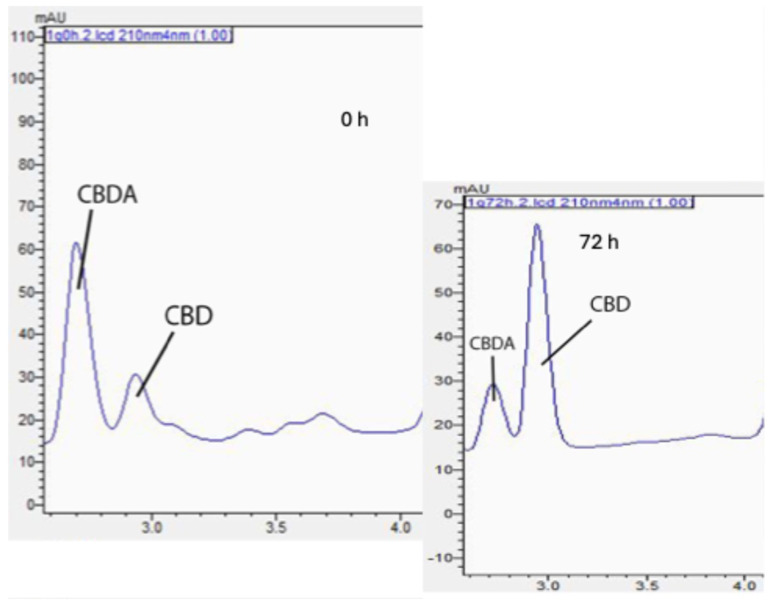
Chromatographic changes in CBDA and CBD after 72 h of thermal treatment on WSM-MD-GA 1:5 particles.

As the core-to-wall ratio increased from 1:5 to 1:2 and subsequently to 1:1, a corresponding increase in CBD degradation was observed, as shown in Figure 5. This trend is attributable to the diminished proportion of wall material relative to the core compound [13], resulting in a greater exposure of the core compound to thermal conditions. Conversely, compared to their non-encapsulated counterpart, these encapsulated formulations enhanced CBD retention, reaching more than twice the amount, thereby emphasizing the advantageous impact of encapsulation technology in preserving cannabinoid integrity against heat [14].

### 3.3. Photo-Stability

Throughout a 72 h duration, the protein-based WSM-HBP-GA formulation demonstrated the greatest resistance to photodegradation, highlighting the protective capabilities of proteins against light exposure. This phenomenon aligns with findings from Zhang et al. [4], where whey protein provided additional protection against UV light by delaying the photodegradation of encapsulated compounds beyond five days of storage. Such effects can be attributed to the proteins’ capacity to reflect or absorb light, with Antosiewicz and Shugar (2016) [15] noting that amino acids possessing aromatic side chains, such as tyrosine and tryptophan, harbor conjugated double bonds, which play a significant role in ultraviolet light absorption. Conversely, Zhu et al. (2021) [16] documented an experiment involving three different protein-encapsulated samples of vitamin A exposed to UV light, which yielded less than 20% recovery of the vitamin A content, indicating that a protein shell alone may not suffice in providing substantial UV protection.

Formulation WSM-MD-GA 1:1 was notably the most susceptible to UV-induced degradation over 72 h, with over 80% of its core content being compromised. This vulnerability can be directly linked to the core-to-wall ratio, with increased concentrations of core compound correlating with increased degradation rates due to the reduced efficacy of the encapsulating material. Similar trends were observed in thermal stability studies, highlighting the pivotal role of core-to-wall ratios in protecting against environmental stressors.

Furthermore, the rate of degradation varied across different formulations, with WSM-HBP-GA 1:5 exhibiting the lowest rate of core compound loss between 24 and 48 h (only 3% loss), while the 1:2 and 1:1 WSM-MD-GA particles displayed the highest degradation rates within the initial 24 h, losing over 50% of their core content. In contrast, non-encapsulated CBD experienced significant degradation, with an 81% reduction within the first 72 h. In an analysis conducted by Fraguas-Sanchez et al. [17], a reduction of 20% in total free CBD content was recorded over 72 h, consequent to ultraviolet (UV) light exposure. These findings emphasize the significant impact of light, particularly UV radiation, on the stability of CBD, highlighting its susceptibility to photodegradation.

## 4. Materials and Methods

### 4.1. Materials

Industrial hemp (*Cannabis sativa* L.) biomass was acquired from Eufloria Medical (Antioch, TN, USA). ∆8-THC, ∆9-THC, cannabinol (CBN), tetrahydrocannabivarin (THCV), cannabidiol (CBD), cannabigerol (CBG), cannabigerolic acid (CBGA), cannabidivarin (CBDV), cannabidolic acid (CBD-A), cannabichromene (CBC), and tetrahydrocannabinolic acid (THCA) standard solutions (1 mg/mL in methanol) were acquired from Cayman Chemical (Ann Arbor, MI, USA). HPLC grade solvents, including acetonitrile and formic acid, were acquired from VWR (Suwanee, GA, USA). All the other reagents such as ethanol, hexane, sodium hydroxide, and hydrogen chloride were obtained from Fisher Scientific (Waltham, MA, USA).

Water soluble mucilage (WSM) and hemp biomass protein (HBP) were extracted and prepared in the lab. WSM was prepared following the methods by Bao et al. 2023 [10], and HBP was prepared using the isoelectric isolation method as described by Julakanti (2023) [18]. HBP was extracted at the solid-to-liquid ratio of 1:15 (*w*/*w*) with milli-Q water. The extraction was adjusted to pH 4.5 and conducted at 80 °C + 0.5 °C with constant stirring for 2 h, followed by centrifugation at 8000× *g* for 10 min (Sorvall ST 16R, Thermo Scientific, Waltham, MA, USA).

### 4.2. Methods

#### 4.2.1. Comparison of Cannabinoid Extraction Solvents by Dynamic Maceration

CBD extraction was adopted from the existing methodology [6] to compare the extraction efficiency of ethanol and hexane. Briefly, hemp biomass was grinded into fine powder to pass through 250 µm sieves. The hemp biomass powder was extracted at a solid-to-liquid ratio of 1:40 for both ethanol and hexane. The extraction was placed at room temperature (25 °C + 0.5 °C) with constant stirring for 15 min, followed by centrifugation at 8000× *g* for 10 min (Sorvall ST 16R, Thermo Scientific). The supernatant was collected, and the residue was extracted at the same condition twice. The supernatants were combined, and the final volume was adjusted to 50 mL accurately. The sample was filtrated with a 0.45 µm syringe filter before being subjected to HPLC analysis.

#### 4.2.2. Evaporation of Supernatant

The supernatant collected from the above extraction was mixed with coconut oil at a ratio of 1:2.6, followed by evaporation using a rotary evaporator (Buchi Rotavapor R-100, New Castle, DE, USA) with the water bath set to 40 °C until solvents were absent. The condensed supernatant was collected and stored at −20 °C for further applications.

#### 4.2.3. CBD Profiling Utilizing HPLC

The HPLC analysis method by Brighenti et al. [6] was adopted with modifications on a Shimadzu Prominence XR UHPLC (Shimadzu Scientific Instruments, Columbia, MD, USA) which included two Shimadzu LC-20ADXR pumps, a SIL-20ACXR autosampler, a CTO-20 A column oven, and a SPD-M20A photo diode array (PDA) UV–VIS detector. Chromatographic separation was achieved with an Ascentis Express C18 column (150 nm × 3.0 mm I.D. × 2.7 µm, Supelco, Bellefonte, PA, USA). The mobile phases contained 0.1% formic acid in both (A) water and (B) acetonitrile. The gradient elution was modified as the following: 0–4 min 70% B, 4–8.50 min 75% B, 8.50–12 min 95% B, 12–18 min 70% B. The flow rate was 0.4 mL/min. The column temperature was set to 30 °C. The injection volume was 10 µL. The UV/DAD parameters were measured at 190–600 nm, while chromatograms were acquired at 210 nm and 220 nm. Injection of samples was performed in triplicates to ensure the reliability of the results. Profiling of the extracted CBD compounds was identified and quantified according to their corresponding retention times and generated standard curves. The following 11 cannabinoids were used as standards: ∆8-tetrahydrocannabinol (∆8-THC), ∆9-tetrahydrocannabinol (∆9-THC), cannabinol (CBN), tetrahydrocannabivarin (THCV), cannabidiol (CBD), cannabigerol (CBG), cannabigerolic acid (CBGA), cannabidivarin (CBDV), cannabidolic acid (CBD-A), cannabichromene (CBC), and tetrahydrocannabinolic acid (THCA) standard solutions (1 mg/mL in methanol). Calibration curves were generated using the concentrations ranged from 0.35 to 10 µg/mL.

#### 4.2.4. Encapsulation of CBD Using Natural Polymers by Freeze-Drying

Preparation of wall materials: Wall material formulation was adopted from Bao et al. [10], where WSM, MD, and GA were weighed, mixed at the ratio of 1:2:5 (*w*/*w*/*w*) as wall material base solutions, and dispersed in water at 70 °C for 1 h with constant stirring.

Preparation of emulsion: The mixture of CBD oil was slowly added to the solution of wall materials at the core to wall ratio of 1:5, 1:2, and 1:1 (*w*/*w*), respectively. The mixture was homogenized for 5 min using Polytron at 11,000 rpm (PT 10-35 GT, Thomas Scientific, Swedesboro, NJ, USA) followed by high-pressure homogenization using a microfluidizer at 15,000 psi (M110P, Microfluidics International Corporation, Westwood, MA, USA). The freshly prepared emulsion was frozen overnight at −20 °C and subjected to freeze-drying (Labconco Corporation, Kansas City, MO, USA). The emulsion was freeze-dried for 36 h and grounded into powder to pass through 425 μm sieves. The powders were stored in tightly capped containers at 4 °C in fridge for further analysis. Similarly, BP was used as a wall ingredient to replace the maltodextrin in the previous formula.

#### 4.2.5. Effect of Thermal Stability

A method by Sanchez et al. [17] was adopted for the thermal stability study of CBD with slight modifications. In short, 0.1 g of CBD, both encapsulated and non-encapsulated, were allocated into individual weighing dishes and exposed to an oven maintained at 70 °C. Each group was retrieved at 0, 24, 48, and 72 h intervals. A portion of 25 mg of each sample were removed and mixed with 5 mL of acetonitrile, stirred overnight or until dissolved and centrifuged at 1000 rpm for 2 min (Sorvall ST 16R, Thermo Scientific). The supernatant was filtered using a 0.45 um syringe filter and subjected to HPLC analysis.

#### 4.2.6. Effect of Photo Treatment

A method by Sanchez et al. [17] was adopted with slight modifications. Briefly, 0.1 g of samples were allocated into individual weighing dishes and exposed to a UV chamber emitting light at 365 nm (UVa) (ENF-260C, Spectroline, Melville, NY, USA) for 72 h in ambient temperature (25 °C ± 0.5 °C). Each group was retrieved at 0, 24, 48, and 72 h intervals. A total of 25 mg of each sample were removed and mixed with 5 mL of acetonitrile, stirred overnight or until dissolved and centrifuged at 1000× *g* for 2 min (Sorvall ST 16R, Thermo Scientific). The supernatant was filtered using a 0.45 um syringe filter and subjected to HPLC analysis.

### 4.3. Statistical Analysis

Experiments were performed in triplicate. The data obtained from these experiments were analyzed by one-way analysis of variance [19] using GraphPad Prism 10 (Ver. 10.3.1, Boston, MA, USA) to see if there were significant differences between any of the groups. For all data statistically significant (*p* < 0.05), Tukey’s HSD test (post ANOVA comparison of multiple means) was performed to determine any differences between treatments. These data are presented as means ± and standard deviation (STDEV).

## 5. Conclusions

In summary, the extraction of cannabinoids through DM revealed that ethanol yields higher concentrations of CBD and CBDA compared to hexane, establishing ethanol as the solvent of choice for subsequent extractions. Specifically, the extraction via ethanol resulted in a yield of 0.11% ± 0.10 CBD per gram of biomass.

During the conducted stability assessments, formulation WSM-HBP-GA 1:5 exhibited superior resilience against UV-induced degradation, whereas the WSM-MD-GA 1:5 formulation demonstrated optimal protection against thermal degradation over 72 h. Incorporating protein within the particle formulation enhanced protection against photodegradation, attributed to amino acids with aromatic side chains capable of absorbing UV radiation. However, this protein formula offered minimal defense against thermal degradation. The comparative analysis between encapsulated and non-encapsulated CBD highlights the significant role played by the wall material and the core-to-wall ratio in alleviating degradation induced by environmental stressors. These findings highlight the volatility of cannabinoids and the profound impact of environmental factors, such as temperature and light, on their stability. Future experiments will delve into the in vivo controlled release effects of WSM-HBP-GA particles to observe the influence of hemp biomass protein on a particle under physiological conditions.

## Figures and Tables

**Table 1:** Comparative analysis of cannabinoid yields per gram of hemp biomass using different solvents in dynamic maceration extraction.

Cannabinoid	DM Solvent	% in Biomass
CBDA	Hexane	1.06 ± 0.04 ^a^
CBDA	Ethanol	1.83 ± 0.00 ^b^
CBD	Hexane	0.08 ± 0.04 ^A^
CBD	Ethanol	0.11 ± 0.10 ^B^
∆9-THC	Hexane	0.30 ± 0.04

Values are presented as the mean ± STDEV of the mean (SEM) values (n = 3). ^a,b,A,B^ There was significant difference between data with the different letters (*p* < 0.05).

## Data Availability

The original contributions presented in this study are included in the article. Further inquiries can be directed to the corresponding author.

## Abbreviations

The following abbreviations are used in this manuscript:

CBDV	Cannabidivarin
CBG	Cannabigerol
CBGA	Cannabigerolic acid
CBDA	Cannabidiolic acid
CBD	Cannabidiol
THCV	Tetrahydrocannabivarin
CBN	Cannabinol
delta-9-THC	Delta-9-tetrahydrocannabinol
delta-8-THC	Delta-8-tetrahydrocannabinol
CBC	Cannabichromene
THCA	Tetrahydrocannabinolic acid
DM	Dynamic Maceration
SFE	Supercritical Fluid Extraction
UAE	Ultrasonic-Assisted Extraction
MAE	Microwave-Assisted Extraction
GA	Gum Arabic
WSM	Water Soluble Yellow Mustard Mucilage
MD	Maltodextrin
HBP	Hemp Biomass Protein
HPLC	High Performance Liquid Chromatography
